# The presence of Merkel cell carcinoma polyomavirus is associated with a distinct phenotype in neoplastic Merkel cell carcinoma cells and their tissue microenvironment

**DOI:** 10.1371/journal.pone.0232517

**Published:** 2020-07-20

**Authors:** María-Dolores Mendoza, Carlos Santonja, Carmen Gonzalez-Vela, Angel Concha, Nicolás Iglesias Pena, Eva-Maria Andrés-Esteban, Jose Pedro Vaque, Laura Cereceda, Raquel Pajares, H. Kutzner, Luis Requena, Miguel A. Piris

**Affiliations:** 1 Department of Dermatology, Fundación Jiménez Díaz, Madrid, Spain; 2 Department of Pathology, Fundación Jiménez Díaz, Madrid, Spain; 3 Department of Pathology, HUMV, Santander, Spain; 4 Department of Pathology, Estructura Organizativa de Xestión Integrada de A Coruña, Coruña, Spain; 5 Department of Dermatology, Estructura Organizativa de Xestión Integrada de A Coruña, Coruña, Spain; 6 Universidad Rey Juan Carlos, PBM.idiPAZ, Madrid, Spain; 7 IDIVAL, Universidad de Cantabria, Santander, Spain; 8 CIBERONC, Madrid, Spain; 9 Dermatopathologie Friedrichshafen, Friedrichshafen, Germany; Virginia Commonwealth University, UNITED STATES

## Abstract

**Aims:**

Merkel cell carcinoma (MCC) is an aggressive primary neuroendocrine tumor of the skin, associated with Merkel cell polyomavirus (MCPyV) in 49–89% of cases, depending on the country of origin and the techniques of detection. The presence of MCPyV defines heterogeneity in MCC; MCPyV-negative cases bear a much higher mutational load, with a distinct ultraviolet signature pattern featuring C > T transitions, as a consequence of exposure to ultraviolet light radiation. MCC stroma has not been thoroughly studied, although MCC patients benefit from therapy targeting PD1/PDL1.

**Methods and results:**

In this study, using Tissue Microarrays and immunohistochemistry, we have analyzed a series of 219 MCC cases in relation to the presence of MCPyV, and confirmed that the presence of MCPyV is associated with changes not only in the neoplastic cells, but also in the composition of the tumor stroma. Thus, MCPyV, found in 101/176 (57,4%) analyzable cases, exhibits changes in its tumor morphology, the density of the inflammatory infiltrate, the phenotype of the neoplastic cells, and the cell composition of the tumor stroma. MCPyV presence is negatively correlated with a higher level of p53 expression, and associated with a very high frequency (86%) of HLA-I expression loss, a higher apoptotic index, and a stroma richer in T-cells, cytotoxic T-cells, macrophages, PDL1-positive macrophages, and B-cells.

**Conclusions:**

Our findings provide evidence of the basic heterogeneity of MCC, supporting the hypothesis that the presence of MCPyV may induce a rich inflammatory response, which is at least partially avoided through loss of HLA-I antigen expression. On the other hand, MCPyV-negative cases show a much higher frequency of stronger p53 expression and, probably, p53 alterations.

## Introduction

Merkel cell carcinoma (MCC) is an aggressive primary neuroendocrine tumor of the skin [1, 2]. It is a rare neoplasm, but more than one-third of patients die of the disease and the case-fatality rate is much higher than that of primary cutaneous melanoma [2]. Dramatic increases in the incidence of, and mortality from, MCC have been described in several countries [3].

MCC is associated with Merkel cell polyomavirus (MCPyV) in 49–89% of cases, depending on the country of origin and the sensitivity of the techniques used. MCPyV-negative cases, in contrast to positive ones, bear a much higher mutational load and have a distinct ultraviolet (UV) signature pattern, featuring C > T transitions, as a consequence of exposure to UV radiation [2, 4–7]. Viral genes are known to play a role in the pathogenesis of MCC, whereby MCPyV viruses express large, small and 57 kDa T antigens that have the potential to inhibit retinoblastoma (RB) activity through the action of large T antigens, and to promote MCC tumorigenesis. Small T (ST) antigen functionally inactivates TP53 by increasing the expression of MDM2 [8].

The crucial role of cellular immunity in MCC development and progression, and the findings of studies of the tumoral microenvironment provided the rationale for immunotherapy [5].

The presence of MCPyV has been associated with changes in tumoral cells. MCPyV-negative cases carry a higher mutational load, featuring mutations involving TP53, RB and other key genes and pathways [4–7, 9]. Relation between the presence of MCPyV and the tumor stroma has been previously investigated, where a relation between the presence of MCPyV and a higher number of T-cells and macrophages was found [10–13]. Previous investigators have also demonstrated that the localization, intensity and phenotype of the T-cells are associated with differences in survival probability [14, 15].

We report the analysis of a large series of MCC cases to assess whether the presence of MCPyV is associated with changes not only in the neoplastic cells but also in the composition of the tumor stroma, in an attempt to understand better the complex relationship between the virus, tumoral cells and the tumor stroma. For this purpose we have analyzed a large series of 219 cases using tissue microarrays to improve the consistency of the multiple immunohistochemistry techniques performed. Markers identifying different subpopulations of macrophages and lymphocytes were used, together with antibodies recognizing the presence of MCPyV, TP53, PDL1, proliferation, apoptosis or the expression of histocompatibility antigens by the neoplastic cells.

The data obtained were used to determine whether the presence or absence of MCPyV identifies essential differences in the MCC pathogenesis and interaction between the stroma and the neoplastic cells

## Material and methods

Our study involves 219 cases, collected between 1995 and 2018, that were initially diagnosed in the following clinical centers: Fundación Jimenez Díaz (FJD), Madrid; Hospital Virgen de la Salud (HVS) Toledo; Complejo Hospitalario Universitario de A Coruña (CHUAC); Complejo Hospitalario Universitario de Vigo (CHUVI); Fundación Instituto Valenciano de Oncología, Valencia; Hospital Universitario Marqués de Valdecilla (HUMV); and the Dermatohistopathology Laboratory, Friedrichshafen (Germany)

### Ethics declaration

All human samples used in this study were collected following the Declaration of Helsinki protocols. We kept the original records under specific restricted conditions to fulfill all current legal requirements. All processes were approved and conducted in adherence to the specific recommendations of the local CEIC (Comité Ético de Investigación Clínica, Fundación Jiménez Díaz, Madrid). (Approval code PIC075-18_FJD). Follow-up clinical data were only available for a smaller group of cases.

I confirm that all data and samples were fully anonymized before we accessed them and that the IRB waived the requirement for informed consent.

Diagnoses were centrally reviewed and confirmed after evaluating morphology and immunostaining for the following markers: CK20, CK7, CAM5.2, chromogranin A, CD56, neuron-specific enolase, synaptophysin and TTF-1. The diagnosis was confirmed in 205 cases for which suitable samples were available for study.

### Immunohistochemistry

Histological slides were reviewed by two of the authors (CS, MP), who have experience in MCC diagnosis, in order to choose representative areas of tumors with which to build tissue microarrays (TMAs). Representative areas for the TMA were selected taking into account the histological variables described in results. For each donor, two 1-mm cores of tissue were collected from the paraffin block using a TMA-I manual tissue arrayer (Beecher Instruments, Sun Prairie, WI). Cores were then arranged in five TMA paraffin blocks to construct the microarray. Cores were selected from areas corresponding to both the center of the tumor and the periphery, in order to allow a wider view of the spatial distribution of the neoplastic cells. Whenever detected, tumor areas with inflammatory infiltrate were selected.

TMA slides were stained using the Avidin–Biotin–Immunoperoxidase technique (LSAB; Dako, Glostrup, Denmark). Antigens were retrieved by incubating the samples with citrate buffer at pH 6.0 and heating in an autoclave (3 min, 1.5 atm). Diaminobenzidine was used as a chromogen and sections were counterstained with hematoxylin.

Each TMA was stained with the following antibodies:

- MCPyV: CM2B4
- P53, PD1, PDL1, FOXP3, ROR gamma, CD20, PAX5, CD4, CD8, CD3, granzyme, perforin, active caspase 3, TIA1, CD68, CD163, CK20, CD56, synaptophysin, chromogranin, Ki 67, HLA I, HLA II, ALK and EZH2

Appropriate positive and negative controls were performed in parallel. Details of specific antibodies and controls are shown in S1 Table. A basic description of the flow chart in described in the S1 Fig.

Immunostaining evaluation and scoring were performed independently by two pathologists (CS, MP). For the markers expressed by the neoplastic cells, the results were scored according to the following semiquantitative criteria: negative/low: <10% positive cells; intermediate: 10–50% positive cells; high: >50% positive cells. Quantification of the results was adapted to the staining patterns of each antibody. Thus, Ki67 was scored only as intermediate or high (using a threshold of 50%), while p53 and MCPyV were scored as positive or negative. MCPyV was considered to be positive if there were >10% positive cells.

Thresholds for the stromal cells were dependent on their relative abundance, as shown in the accompanying figures.

### Statistical analysis

Continuous values were summarized as means, and maximum and minimum values. Distributions of categorical variables were summarized as absolute frequencies and percentages. Chi-square contingency tests were performed to analyze the differences in stromal and tumoral marker expression between the MCPyV-positive and MCPyV-negative cases.

## Results

The basic clinical features of the cases included in this series are described in Table 1 Diagnostic clinical data were available from 196 patients. Mean age was 78,7 yrs (range 48–105). Ratio male/female was 81(41,3%)/115 (58,7%). It was possible to ascribe an AJCC stage (8^th^ edition) to 75 patients. Death attributable to the tumor was concluded for 29/64 (45.3%) patients.

**Table 1:** Main clinical features.

Age (median)	78.7 yrs (range 48–105)
Sex (N:196)	81 Male (41,3%) /115 Female (58,7%)
Site (N:168)	Head and neck: 76 (45,2%)
	Trunk: 7 (4,2%)
	Upper extremities: 23 (13,7%)
	Lower extremities: 59 (35,2%)
	Lymph node: 3 (1,7%)
AJCC 8^th^ edition Clinical stage(N:75)	I: 14 (18,7%)
	II: 21 (28%)
	III: 21 (28%)
	IV: 19 (25,3%)
Immunosuppression: HIV, autoimmune disease, transplant, cancer (N:97)	18 (18,5%)
Death by the tumour*: (N:64)	29 (45,3%)

* Full clinical diagnostic and follow-up information was only available in 64 cases

Basic histological features are described in Tables 2 and 3, as estimated in whole-tumor sections. The intensity of the intratumoral inflammatory infiltrate was scored as low, intermediate, or high, in accordance with the proposal described in the ICCR Merkel Cell Carcinoma Histopathology Reporting Guide (http://www.iccr-cancer.org/datasets/datasets-under-consultation). A high inflammatory infiltrate was observed in 27% of cases, while an intermediate infiltrate was found in 33% of samples, with 40% cases exhibiting a low proportion of inflammatory cells.

**Table 2:** Basic histological variables.

Pattern (N:144)	Nodular 112 (77.8%)	Trabecular 19 (13.2%)	Mixed 13 (9%)
Lymphocytic infiltrate (N:134)	Low 54 (40.3%)	Intermediate 44 (32.8%)	High 36 (26.9%)
Level of infiltration (N:76)	Dermis 22 (28.9%)	Hypodermis 44 (57.9%)	Muscle 10 (13.2%)

**Table 3:** Basic histological variables.

VARIABLES	PRESENCE
Vascular invasión	34/133 (25.6%)
Necrosis	56/132 (42.4%)
Ulcer	27/131 (20.6%)
Regression	97/205 (47.3%)

Intratumoral fibrosis was recognized by the presence of areas of intratumoral scars, paucicellular areas within the tumor, which are quite frequently associated with areas of high-density lymphocytic infiltration. High-density lymphoid infiltrate and/or intratumoral fibrosis were identified in 97/205 (47.3%) cases (S2 Fig), both features being taken as possible evidence of tumor regression. In this series, there was no relation between the intensity of the lymphoid infiltrate and the survival probability.

The main IHC markers for the neoplastic cells, measured in TMAs, are listed in Table 4. MCPyV positivity was found in 101 of the 176 (57.3%) cases considered to be analyzable. Strong uniform p53 expression by the neoplastic cells was observed in 40/180 (22.2%) cases. PAX5 was found to be expressed by the neoplastic cells in 59% of the samples. Most cases exhibited ALK expression, with only 15/174 (8.6%) cases recorded as negative. HLA-I expression was partially or completely lost in most cases, with only 49/180 (27.2%) cases showing homogeneous staining. HLA-II expression was only found in 2/180 (1.1%) cases. Active caspase 3 was used to measure the level of apoptosis, with only 4% of cases yielding negative results. PDL1 expression by the neoplastic cells was found in 22/179 (12.3%) cases. CD56 expression was found in the tumoral cells of 144/169 (85.2%) cases.

**Table 4:** Main IHC markers for the neoplastic cells.

MARKERS		NEGATIVE/LOW	INTERMEDIATE	POSITIVE
Cytokeratin	CK20 (N:182)	27 (14,8%)	NA	155 (85.2%)
Neuroendocrine markers	Synaptophysin (N:164)	9 (5.5%)	31 (18.9%)	124(75.6%)
	Chromogranin (N:184)	20 (10.9%)	49 (26.6%)	115(62.5%)
	CD56 (N: 175)	25 (14.3%)	41 (23.4%)	109 (62.3%)
MCPyV	MCPyV (N:176)	75 (43%)	NA	101 (57%)
P53	P53 (N:180)	140 (77.8%)	NA	40 (22.2%)
Oncogenes	ALK (N:174)	15 (8.6%)	39 (22.4%)	120 (69%)
	EZH2 (N:164)	4 (2.3%)	12 (6.9%)	158 (90.8%)
Proliferation	Ki67 (N:138)	NA	103 (74.6%)	35 (25.4%)
Apoptosis	Active Caspase 3 (N:137)	6 (4.3%)	41 (30%)	90 (65.7%)
Immune regulation	HLA-I (N:180)	78 (43.3%)	53 (29.4%)	49 (27.3%)
	HLA-II (N:180)	178 (98.8%)	1 (0.6%)	1 (0.6%)
	PDL1 tumor (N: 179)	157 (87.7%)	NA	22 (12.3%)
PAX5	PAX 5 (183)	75 (41%)	NA	108 (59%)

NA: Not available. Most markers were read as Negative-Low/Intermediate/Positive. A few markers were just dichotomized into Negative/Positive (MCPyV, P53, PDL1), or Intermediate/Positive (Ki67)

The cell composition of the neoplastic stroma was analyzed, taking into account the presence and phenotype of lymphocytes, myeloid cells and macrophages (Table 5)

**Table 5:** Main IHC markers for the stromal cells.

MARKERS		NEGATIVE/LOW	INTERMEDIATE	POSITIVE
MACROPHAGES	CD68 (N: 178)	5 (2.8%)	124 (69.7%)	49 (27.5%)
	CD163 (N:180)	12 (6.7%)	99 (55%)	69 (38.3%)
	PDL1 (N:176)	83 (47.2%)	NA	93 (52.8%)
CYTOTOXIC	Granzyme (N:178)	82 (46.1%)	73 (41%)	23 (12.9%)
	Perforin (N:110)	91 (82.7%)	3 (2.7%)	16 (14.6%)
	TIA1 (N:177)	41 (23.2%)	93 (52.5%)	43 (24.3%)
T-CELLS	CD3 (N:175)	64 (36.6%)	60 (34.3%)	51 (29.1%)
	CD4 (N:181)	84 (46.4%)	NA	97 (53.6%)
	CD8 (N:180)	66 (36.7%)	NA	114 (63.3%)
	FOXP3 (N:177)	92(52%)	9 (5.1%)	76 (42.9%)
	ROR gamma (N:180)	153 (85%)	4 (2.2%)	23 (12.8%)
	PD1 (N:176)	95 (54%)	NA	81(46%)
B-CELLS	CD20 (N:177)	128 (72.3%)	NA	49 (27.7%)

NA: Not available. Most markers were read as Negative-Low/Intermediate/Positive. A few markers were just dichotomised into Negative/Positive (PDL1 stroma, CD4, CD8, CD20, PD1)

Specific thresholds were used for each marker depending on the relative presence. A conspicuous T-cell infiltrate was seen in roughly two-thirds of the cases, with frequent cytotoxic T-cells, defined by the expression of TIA1, perforin or granzyme B. The vast majority of cases featured some macrophages in the stroma (CD68 or CD163), with roughly half of the cases having PDL1-positive macrophages. CD3+ CD4+ T-cells were also present in a significant number of cases (97/181) (53.6%), and B-cells (identified with CD20) were present in two-thirds of the cases. Presence of specific subpopulations of T-cells were also identified: FOXP3 regulatory T-cells were present in 85/177 (48.0%) cases, while TH17 T-cells recognized by the antibody ROR-GT were found in 27/180 (15.0%) cases.

### Relationship with MCPyV expression

**Fig 1:**
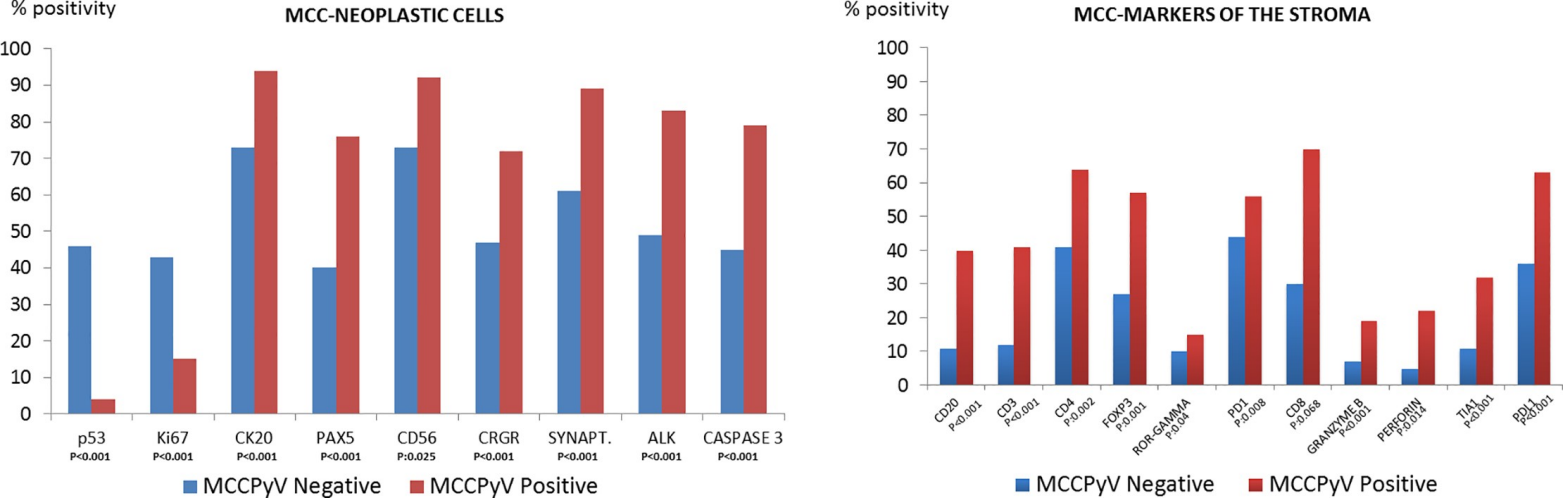
MCC-neoplastic cells.

**Fig 2:**
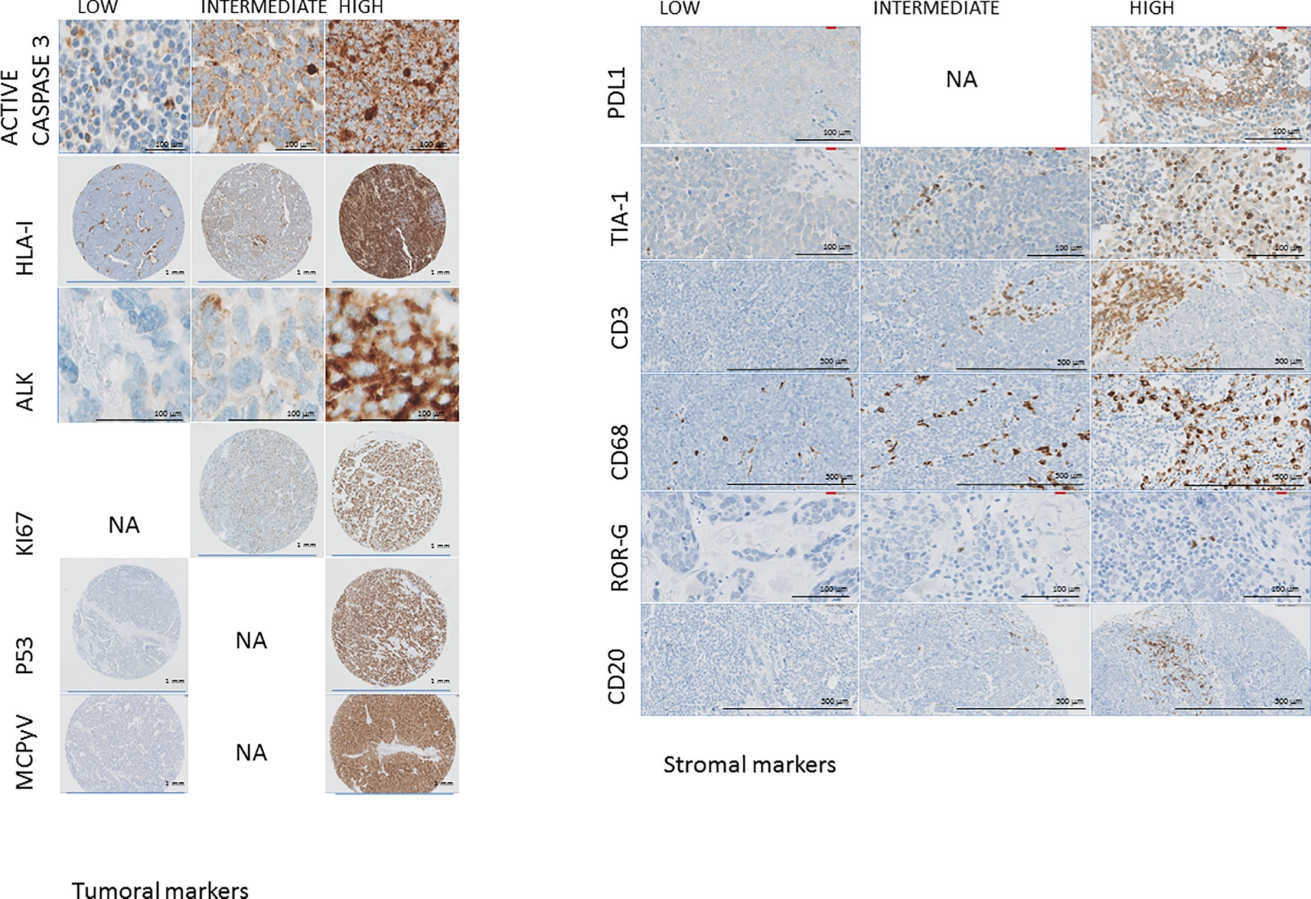
Differences in the expression of the most significant markers. **A:** Neoplastic cells: Caspase 3, HLA-I, ALK, KI67, P53. **B:** Tumor stroma: PDL1, TIA1, CD3, CD68, ROR GAMMA, CD20.

Histological and immunohistochemical markers in MCPyV-positive and MCPyV-negative cases were compared. The significant findings are presented in Table 6 and illustrated in Figs 1 and 2. The most important of these are described below.

**Table 6:** Comparison between MCPyV-positive and negative cases.

	MARKERS	TOTAL	MCPyV POSITIVE	MCPyV NEGATIVE	SIGNIFICANCE
Morphology	Combined histology Pure MCC N:124	Combined: 19 (15.3%)	2 (2.9%)	17 (31.8%)	P<0.001, Chi2:19.25
		Pure MCC: 105 (84.7%)	68 (97.1%)	37 (68.5%)	
	Lymphoid infiltration N:127	Low: 53(41.7%)	23(32.4%)	30(53.6%)	P:0.013, Chi2: 8.64
		Intermediate: 41(32.3%)	23(32.3%)	18(32.1%)	
		High: 33 (26%)	25(35.2%)	8 (14.3%)	
	UIceration N:124	Presence: 26 (21%)	4 (5.9%)	22 (39.3%)	P<0.001, Chi2:20.68
		Absence: 98 (79%)	64(94.1%)	34 (60.7%)	
	Elastosis N:118	Presence 65 (55.1%)	27(41.5%)	38 (71.7%)	P:0.001, Chi2: 10.73
		Absence: 53 (44.9%)	38(58.5%)	15 (28.3%)	
IHC neoplastic cells	CK20 N:172	Positive: 146(84.8%)	92(93.9%)	54(73%)	P<0.001, Chi2: 14.36
		Negative/Low: 26 (15.1%)	6 (6.1%)	20(27.0%)	
	P53 N:171	High: 38(22.2%)	4(4.1%)	34(45.9%)	P<0.001, Chi2: 42.48
		Negative/Low: 133(77.8%)	93(95.9%)	40(54.0%)	
	Ki67 N:132	High: 35(26.5%)	12(15.4%)	23(42.6%)	P<0.001, Chi2: 12.12
		Intermediate: 97 (73.5%)	66(84.6%)	31(57.4%)	
	Chromogranin N:171	High: 105(61.4%)	71(71.7%)	34(47.2%)	P<0.001, Chi2: 18.32
		Negative/Low: 19 (11.1%)	3 (3%)	16(22.2%)	
		Intermediate: 47 (27.5%)	25(25.2%)	22(30.6%)	
	Synaptophysin N:152	High: 118(77.6%)	80(88.9%)	38(61.3%)	P<0.001, Chi2: 16.15
		Negative/Low: 6 (4%)	2(2.2%)	4(6.4%)	
		Intermediate: 28 (18.4%)	8 (8.9%)	20(32.3%)	
	ALK N:169	High: 113(68.5%)	79(83.2%)	34(48.6%)	P<0.001; Chi2: 24.96
		Negative/Low: 15 (9.1%)	2 (2.1%)	13(18.6%)	
		Intermediate 37(22.4%)	14(14.7%)	23(32.9%)	
	EZH2 N:166	High: 151(91%)	90(95.7%)	61(84.7%)	P:0.014; Chi2: 8.47
		Negative/Low: 3 (1.8%)	2 (2.1%)	1(1.4%)	
		Intermediate: 12 (7.2%)	2 (2.1%)	10(13.9%)	
	HLA-I N:166	High: 44(26.2%)	13(13.7%)	31(42.5%)	p<0.001; Chi2: 25.04
		Negative/Low: 72(42.9%)	55(57.9%)	17(23.3%)	
		Intermediate: 52(30.9%)	27(28.4%)	25(34.2%)	
	Active Caspase 3 N:127	High: 82(64.6%)	57(79.2%)	25(45.4%)	P<0.001; Chi2: 18.62
		Negative/Low: 6(4.7%)	0 (0.0%)	6(10.9%)	
		Intermediate: 39(30.7%)	15(20.8%)	24(43.6%)	
	PAX5 N:171	High: 103(60.2%)	74(75.5%)	29(39.7%)	P<0.001; Chi2: 22.37
		Negative/Low: 68(39.8%)	24(24.5%)	44(60.3%)	
	CD56 N:169	High: 105 (62.1%)	64(66.7%)	41(56.2%)	P:0.025; Chi2: 7.36
		Negative/Low: 25 (14.8%)	8(8.3%)	17(23.3%)	
		Intermediate: 39 (23.1%)	24(25%)	15(20.6%)	
IHC Tumour stroma	CD3 N:169	High: 48 (28.4%)	39(40.6%)	9(12.3%)	P<0.001; Chi2: 16.33
		Negative/low: 62 (36.7%)	29(30.2%)	33(45.2%)	
		Intermediate: 59 (34.9%)	28(29.2%)	31(42.5%)	
	CD4 N:172	High: 93 (54.1%)	63(64.3%)	30(40.5%)	P:0.002; Chi2: 9.57
		Negative/low: 79(45.9%)	35(25.7%)	44(59.5%)	
	CD20 N:169	High: 46(27.2%)	38(39.6%)	8(11%)	P<0.001; Chi2: 17.15
		Low: 123(72.8%)	58(60.4%)	65(89%)	
	FOXP3 N:165	High: 73 (44.2%)	54(56.8%)	19(27.1%)	P:0.001; Chi2: 14.46
		Negative/Low 87 (52.7%)	39(41%)	48(68.6%)	
		Intermediate: 5 (3%)	2(2.1%)	3(4.3%)	
	ROR gamma N:168	High: 22(13.1%)	15(15.5%)	7(9.9%)	P:0.04; Chi2: 6.4533
		Negative/Low: 142(84.5%)	82(84.5%)	60(84.5%)	
		Intermediate: 4(2.4%)	0 (0%)	4(2.4%)	
	PD1 N:167	High: 78(46.7%)	52(55.9%)	41(44.1%)	P:0.008; Chi2: 7.15
		Negative/Low: 89(53.3%)	26(35.1%)	48(64.9%)	
	CD8 N: 169	High: 108(63.9%)	67(69.8%)	29(30.2%)	P:0.068; Chi2: 3.34
		Negative/Low: 61(36.1%)	29(30.2%)	32(43.8%)	
	CD68 N:169	High: 46(27.2%)	36(37.1%)	10(13.9%)	P:0.001; Chi2: 18.62
		Negative/Low: 5(3%)	1 (1%)	4 (5.6%)	
		Intermediate: 118(69.8%)	60(61.9%)	58(80.9%)	
	Granzyme B N:169	High: 23 (13.6%)	18(18.6%)	5(6.9%)	P<0.001, Chi2:15.24
		Negative/Low: 77(45.6%)	32(33%)	45(62.5%)	
		Intermediate: 69(40.8%)	47(48.4%)	22(30.6%)	
	Perforin N:101	High: 15 (14.8%)	13(22%)	2(4.8%)	P:0.014, Chi2:8.56
		Negative/Low: 83(82.2%)	43(72.9%)	40(95.2%)	
		Intermediate: 3(3%)	3(5%)	0 (0%)	
	TIA1 N:169	High: 39(23.1%)	31(32%)	8(11.1%)	P<0.001; Chi2: 17.04
		Negative/Low: 39(23.1%)	13(13.4%)	26(36.1%)	
		Intermediate 91(53.8%)	53(54.6%)	38(52.8%)	
	PDL1 stroma N: 167	High: 87(52.1%)	60(63.8%)	27(37%)	P:0.001; Chi2: 11.86
		Negative/Low: 80(47.9%)	34(36.2%)	46(63%)	

MCPyV-positive cases showed changes in morphology, the density of the inflammatory infiltrate, the phenotype of the neoplastic cells, and the cell composition of the tumor stroma. Specifically, MCPyV-positive cases had, among other things, a much lower frequency of higher p53 expression (4% *vs*. 46% cases), a higher frequency of ALK expression (83% *vs*. 49%), a more frequent loss of HLA-I expression (86% *vs*. 58%), a higher apoptotic index with more intense expression of active caspase 3) (79% *vs*. 45%), and a stroma richer in T-cells, cytotoxic T-cells, macrophages, PDL1-positive macrophages and B-cells (Table 6).

## Discussion

MCC was originally described as a primary cutaneous neuroendocrine small-cell cancer with aggressive behavior [1]. It was subsequently noted that most MCC cases carried MCPyV, the frequency varying between 49% and 89%, depending on the extent of exposure to UV radiation and the detection technique [16].

In this study, we have detected the presence of MCPyV using the antibody CM2B4, as validated by Moshiri et al [17]. The frequency of MCPyV positivity is consistent with those noted in other studies performed in Southern Europe employing a combination of PCR and IHC [5, 17]. The results confirm that TMAs can be used in the analysis of complex solid tumors; thus, the use of antibodies recognizing diverse cell types can be associated with multiple markers recognizing the expression of genes involved in MCC pathogenesis.

Molecular studies have previously shown that MCPyV-positive cases exhibit a different spectrum of molecular alterations [4, 5, 18, 19], although these studies have generally been of series with relatively small numbers of cases. Our study shows that MCPyV-positive and MCPyV-negative cases differ with respect to most of the markers analyzed here, particularly the higher level of p53 expression revealed by IHC (46% *vs*. 4%), but also the expression of multiple markers. The phenotype of MCPyV-positive cases is characterized by more frequent CK20 expression, a very low frequency of p53 expression, a lower level of Ki67 expression, more frequent neuroendocrine differentiation, and more intense ALK and EZH2 expression. These findings confirm and build on previous observations. It is of particular note the high frequency of HLA-I loss found in this series, a finding that is particularly striking in MCPyV-positive cases (86% *vs*. 43% in the negative cases). These data are in line with previously published data showing that HLA-I loss is observed in a majority of MCC samples, and caused by epigenetic silencing of the antigen presentation machinery [20]. This phenomenon is thought to underlie the regression of metastatic MCC following transfer of polyomavirus-specific T-cells and therapies capable of re-inducing HLA class-I [21, 22].

In this context, it is significant that the expression of active caspase 3 demonstrates that MCPyV-positive cases contain a higher proportion of apoptotic cells than negative cases. The apoptotic index could be interpreted, in this situation, as a surrogate marker of the intensity of the antitumoral immune response.

This study has also included a set or markers that identify some key components of the tumor microenvironment. Bearing in mind the possibilities that immunotherapy has opened up for the treatment of MCC cases, we consider it of particular significance that our study also shows the presence of MCPyV to be associated with differences in the tumor stroma. MCPyV-positive cases are characterized by differences in the proportion of specific T-cell subpopulations and macrophages. Specifically, they have a higher proportion of CD3+, CD4+, FOXP3+, RORgamma+, PD1+ T-cells, and a more cytotoxic phenotype (increase in TIA1, granzyme B, perforin), an increased presence of CD20+, and a higher proportion of CD68+ macrophages (but not of CD163) and PDL1-positive macrophages. These results confirm previously published data [10–15] and extend these observations to the identification of specific T-cell, macrophage and B-cell subsets whose presence is associated with MCPyV. Our data concerning PDL1 expression are largely consistent with those recently published by Walsh and coworkers [11], i.e., PDL1 is mainly expressed by peritumoral macrophages rather than neoplastic cells [13, 23]: in our series, PDL1 expression by neoplastic cells was observed in just 22/179 (12%) cases, a slightly lower proportion than that found by Wehkamp et al and Benigni et al [24, 25]. Our results are basically consistent with those of Miller et al, who showed that MCPyV-positive tumors had a strikingly more clonal intratumoral TCR repertoire than MCPyV-negative tumors [26].

These findings could give support to the interpretation that the presence of MCPyV may induce a rich inflammatory response, which is at least partially evaded through loss of HLA-I antigen expression. The increased level of somatic mutations, as found in the MCPyV-negative cases, could also induce an inflammatory response, albeit with a lower intensity, that may also explain the relatively lower frequency of the loss of HLA-I expression.

The use of tissue microarrays have obvious limitations in the semi-quantitative assessment of different cellular populations; but at the same time improves the reproducibility of the results since basic experimental conditions are much more similar [27]. Data here generated are consistent with previously published data, which is supporting the experimental approach; while at the same time expand the knowledge on the interaction between the MCC cells and the stromal cell subpopulations.

Overall, our study provides strong evidence that MCCs carrying MCPyV have very different features from those of MCPyV-negative cases, and extends previous observations about mutational load [4–7, 9] to the field of stroma cellular composition, thereby implying that MCPyV-positive and MCPyV-negative cases are different tumors in terms of their molecular pathogenesis and prognosis.

## Supporting information

S1 Table Immunohistochemical marker references. (XLSX)

S2 Table (XLSX)

S1 Fig Flow chart for samples tissue and research data obtention. (PPTX)

S2 Fig Intensity of the inflammatory infiltrate scored from low to intermediate and high. Eosinophilic scars are frequently associated with high inflammatory infiltrate.
(PPTX)

## References

[1] Toker C. Trabecular carcinoma of the skin. Arch Dermatol. 1972;105(1):107–10. . 5009611

[2] Becker JC, Stang A, DeCaprio JA, Cerroni L, Lebbe C, Veness M, et al Merkel cell carcinoma. Nat Rev Dis Primers. 2017;3:17077 10.1038/nrdp.2017.77 29072302 PMC6054450

[3] Fitzgerald TL, Dennis S, Kachare SD, Vohra NA, Wong JH, Zervos EE. Dramatic Increase in the Incidence and Mortality from Merkel Cell Carcinoma in the United States. Am Surg. 2015;81(8):802–6. . 26215243 10.1177/000313481508100819

[4] Harms PW, Vats P, Verhaegen ME, Robinson DR, Wu YM, Dhanasekaran SM, et al The Distinctive Mutational Spectra of Polyomavirus-Negative Merkel Cell Carcinoma. Cancer research. 2015;75(18):3720–7. Epub 2015/08/05. 10.1158/0008-5472.CAN-15-0702 26238782 PMC4573907

[5] Gonzalez-Vela MDC, Curiel-Olmo S, Derdak S, Beltran S, Santibanez M, Martinez N, et al Shared Oncogenic Pathways Implicated in Both Virus-Positive and UV-Induced Merkel Cell Carcinomas. The Journal of investigative dermatology. 2017;137(1):197–206. 10.1016/j.jid.2016.08.015 . 27592799

[6] Goh G, Walradt T, Markarov V, Blom A, Riaz N, Doumani R, et al Mutational landscape of MCPyV-positive and MCPyV-negative Merkel cell carcinomas with implications for immunotherapy. Oncotarget. 2016;7(3):3403–15. 10.18632/oncotarget.6494 26655088 PMC4823115

[7] Knepper TC, Montesion M, Russell JS, Sokol ES, Frampton GM, Miller VA, et al The Genomic Landscape of Merkel Cell Carcinoma and Clinicogenomic Biomarkers of Response to Immune Checkpoint Inhibitor Therapy. Clinical cancer research: an official journal of the American Association for Cancer Research. 2019;25(19):5961–71. 10.1158/1078-0432.CCR-18-4159 31399473 PMC6774882

[8] Park DE, Cheng J, Berrios C, Montero J, Cortes-Cros M, Ferretti S, et al Dual inhibition of MDM2 and MDM4 in virus-positive Merkel cell carcinoma enhances the p53 response. Proc Natl Acad Sci U S A. 2019;116(3):1027–32. 10.1073/pnas.1818798116 30598450 PMC6338866

[9] Wong SQ, Waldeck K, Vergara IA, Schroder J, Madore J, Wilmott JS, et al UV-Associated Mutations Underlie the Etiology of MCV-Negative Merkel Cell Carcinomas. Cancer research. 2015;75(24):5228–34. 10.1158/0008-5472.CAN-15-1877 . 26627015

[10] Sihto H, Bohling T, Kavola H, Koljonen V, Salmi M, Jalkanen S, et al Tumor infiltrating immune cells and outcome of Merkel cell carcinoma: a population-based study. Clinical cancer research: an official journal of the American Association for Cancer Research. 2012;18(10):2872–81. 10.1158/1078-0432.CCR-11-3020 . 22467679

[11] Walsh NM, Castonguay MC, Carter MD, Pasternak S, Ly TY, Doucette S, et al Global PD-L1 Signals and Tumor-Infiltrating Lymphocytes: Markers of Immunogenicity in Different Subsets of Merkel Cell Carcinoma and Potential Therapeutic Implications. Am J Dermatopathol. 2019;41(11):819–25. 10.1097/DAD.0000000000001390 . 31634167

[12] Walsh NM, Fleming KE, Hanly JG, Dakin Hache K, Doucette S, Ferrara G, et al A morphological and immunophenotypic map of the immune response in Merkel cell carcinoma. Hum Pathol. 2016;52:190–6. 10.1016/j.humpath.2016.02.002 . 26980039

[13] Lipson EJ, Vincent JG, Loyo M, Kagohara LT, Luber BS, Wang H, et al PD-L1 expression in the Merkel cell carcinoma microenvironment: association with inflammation, Merkel cell polyomavirus and overall survival. Cancer Immunol Res. 2013;1(1):54–63. 10.1158/2326-6066.CIR-13-0034 24416729 PMC3885978

[14] Feldmeyer L, Hudgens CW, Ray-Lyons G, Nagarajan P, Aung PP, Curry JL, et al Density, Distribution, and Composition of Immune Infiltrates Correlate with Survival in Merkel Cell Carcinoma. Clinical cancer research: an official journal of the American Association for Cancer Research. 2016;22(22):5553–63. 10.1158/1078-0432.CCR-16-0392 27166398 PMC5857157

[15] Paulson KG, Iyer JG, Tegeder AR, Thibodeau R, Schelter J, Koba S, et al Transcriptome-wide studies of merkel cell carcinoma and validation of intratumoral CD8+ lymphocyte invasion as an independent predictor of survival. Journal of clinical oncology: official journal of the American Society of Clinical Oncology. 2011;29(12):1539–46. 10.1200/JCO.2010.30.6308 21422430 PMC3082974

[16] Feng H, Shuda M, Chang Y, Moore PS. Clonal integration of a polyomavirus in human Merkel cell carcinoma. Science. 2008;319(5866):1096–100. 10.1126/science.1152586 18202256 PMC2740911

[17] Moshiri AS, Doumani R, Yelistratova L, Blom A, Lachance K, Shinohara MM, et al Polyomavirus-Negative Merkel Cell Carcinoma: A More Aggressive Subtype Based on Analysis of 282 Cases Using Multimodal Tumor Virus Detection. The Journal of investigative dermatology. 2017;137(4):819–27. 10.1016/j.jid.2016.10.028 27815175 PMC5565758

[18] Goh G, Walradt T, Markarov V, Blom A, Riaz N, Doumani R, et al Mutational landscape of MCPyV-positive and MCPyV-negative merkel cell carcinomas with implications for immunotherapy. Oncotarget. 2015 10.18632/oncotarget.6494 . 26655088 PMC4823115

[19] Carter MD, Gaston D, Huang WY, Greer WL, Pasternak S, Ly TY, et al Genetic profiles of different subsets of Merkel cell carcinoma show links between combined and pure MCPyV-negative tumors. Hum Pathol. 2018;71:117–25. 10.1016/j.humpath.2017.10.014 . 29079179

[20] Ritter C, Fan K, Paschen A, Reker Hardrup S, Ferrone S, Nghiem P, et al Epigenetic priming restores the HLA class-I antigen processing machinery expression in Merkel cell carcinoma. Sci Rep. 2017;7(1):2290 10.1038/s41598-017-02608-0 28536458 PMC5442125

[21] Paulson KG, Voillet V, McAfee MS, Hunter DS, Wagener FD, Perdicchio M, et al Acquired cancer resistance to combination immunotherapy from transcriptional loss of class I HLA. Nat Commun. 2018;9(1):3868 10.1038/s41467-018-06300-3 30250229 PMC6155241

[22] Chapuis AG, Afanasiev OK, Iyer JG, Paulson KG, Parvathaneni U, Hwang JH, et al Regression of metastatic Merkel cell carcinoma following transfer of polyomavirus-specific T cells and therapies capable of re-inducing HLA class-I. Cancer Immunol Res. 2014;2(1):27–36. 10.1158/2326-6066.CIR-13-0087 24432305 PMC3888869

[23] Mitteldorf C, Berisha A, Tronnier M, Pfaltz MC, Kempf W. PD-1 and PD-L1 in neoplastic cells and the tumor microenvironment of Merkel cell carcinoma. Journal of cutaneous pathology. 2017;44(9):740–6. 10.1111/cup.12973 . 28569410

[24] Wehkamp U, Stern S, Kruger S, Weichenthal M, Hauschild A, Rocken C, et al Co-expression of NGF and PD-L1 on tumor-associated immune cells in the microenvironment of Merkel cell carcinoma. J Cancer Res Clin Oncol. 2018;144(7):1301–8. 10.1007/s00432-018-2657-x . 29744662 PMC11813430

[25] Benigni P, Guenole M, Bonsang B, Marcorelles P, Schick U, Uguen A. Foci of Programmed Cell Death-Ligand 1 (PD-L1)-positive Tumor Areas With Tumor-infiltrating Leukocytes (TILs) Evocative of a PD-1/PD-L1-related Adaptive Immune Resistance are Frequent in Merkel Cell Carcinoma. Appl Immunohistochem Mol Morphol. 2019 10.1097/PAI.0000000000000792 . 31343994

[26] Miller NJ, Church CD, Fling SP, Kulikauskas R, Ramchurren N, Shinohara MM, et al Merkel cell polyomavirus-specific immune responses in patients with Merkel cell carcinoma receiving anti-PD-1 therapy. J Immunother Cancer. 2018;6(1):131 10.1186/s40425-018-0450-7 30482247 PMC6258401

[27] Hewitt SM. Tissue microarrays as a tool in the discovery and validation of tumor markers. Methods Mol Biol. 2009;520:151–61. 10.1007/978-1-60327-811-9_11 19381953 PMC7297263

