# Supplementary figures and images for: The presence of Merkel cell carcinoma polyomavirus is associated with a distinct phenotype in neoplastic Merkel cell carcinoma cells and their tissue microenvironment
Source: PLoS One. 2020 Jul 20;15(7):e0232517. doi: 10.1371/journal.pone.0232517 (PMC7371188; doi:10.1371/journal.pone.0232517)

## Slide 1
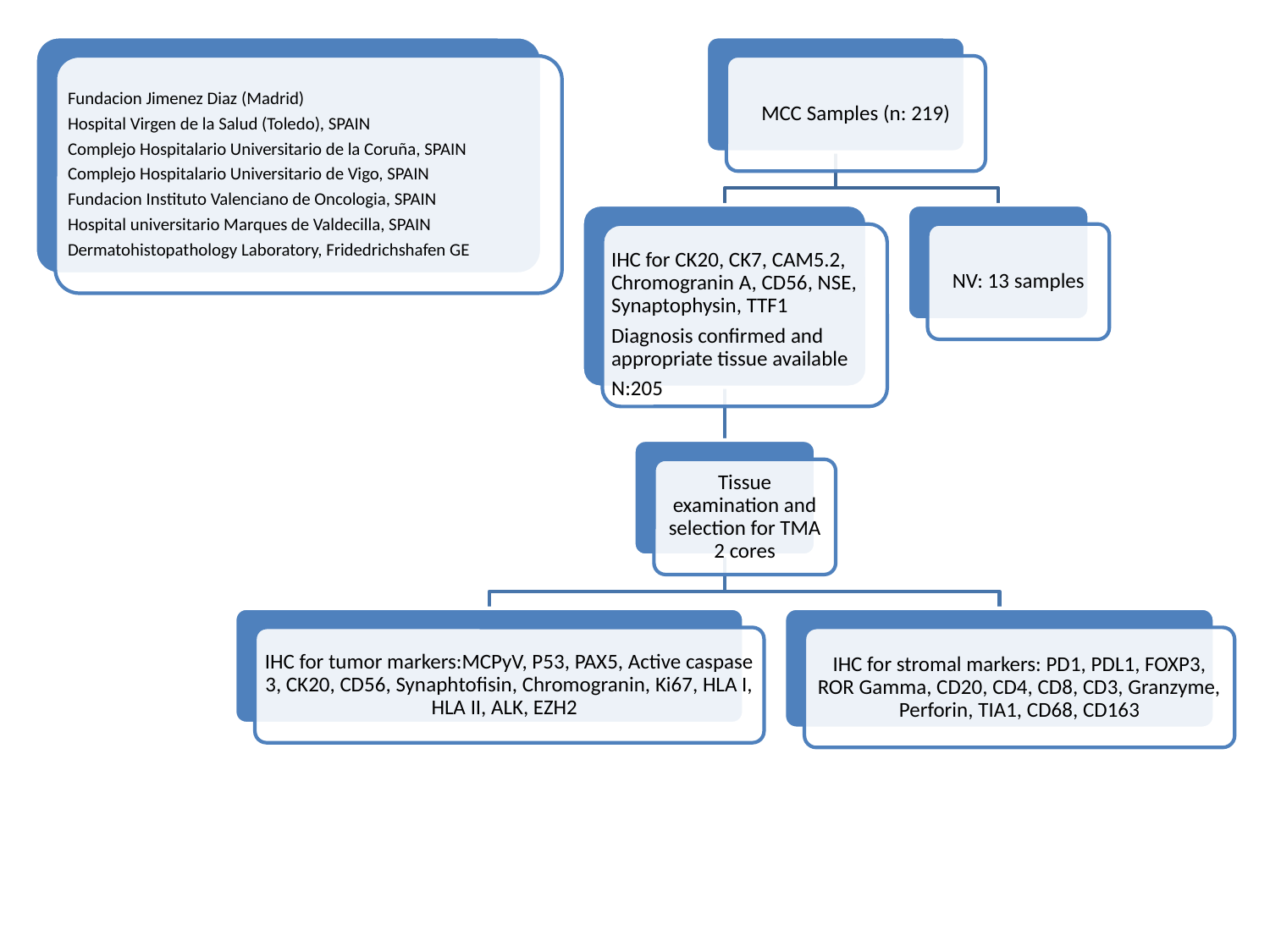

Supplement: S1 Fig — (PPTX) [file pone.0232517.s003.pptx]
