# Supplementary material for: The presence of Merkel cell carcinoma polyomavirus is associated with a distinct phenotype in neoplastic Merkel cell carcinoma cells and their tissue microenvironment
Source: PLoS One. 2020 Jul 20;15(7):e0232517. doi: 10.1371/journal.pone.0232517 (PMC7371188; doi:10.1371/journal.pone.0232517)
Supplement: S2 Fig — Eosinophilic scars are frequently associated with high inflammatory infiltrate. (PPTX) [file pone.0232517.s004.pptx]

## Slide 1
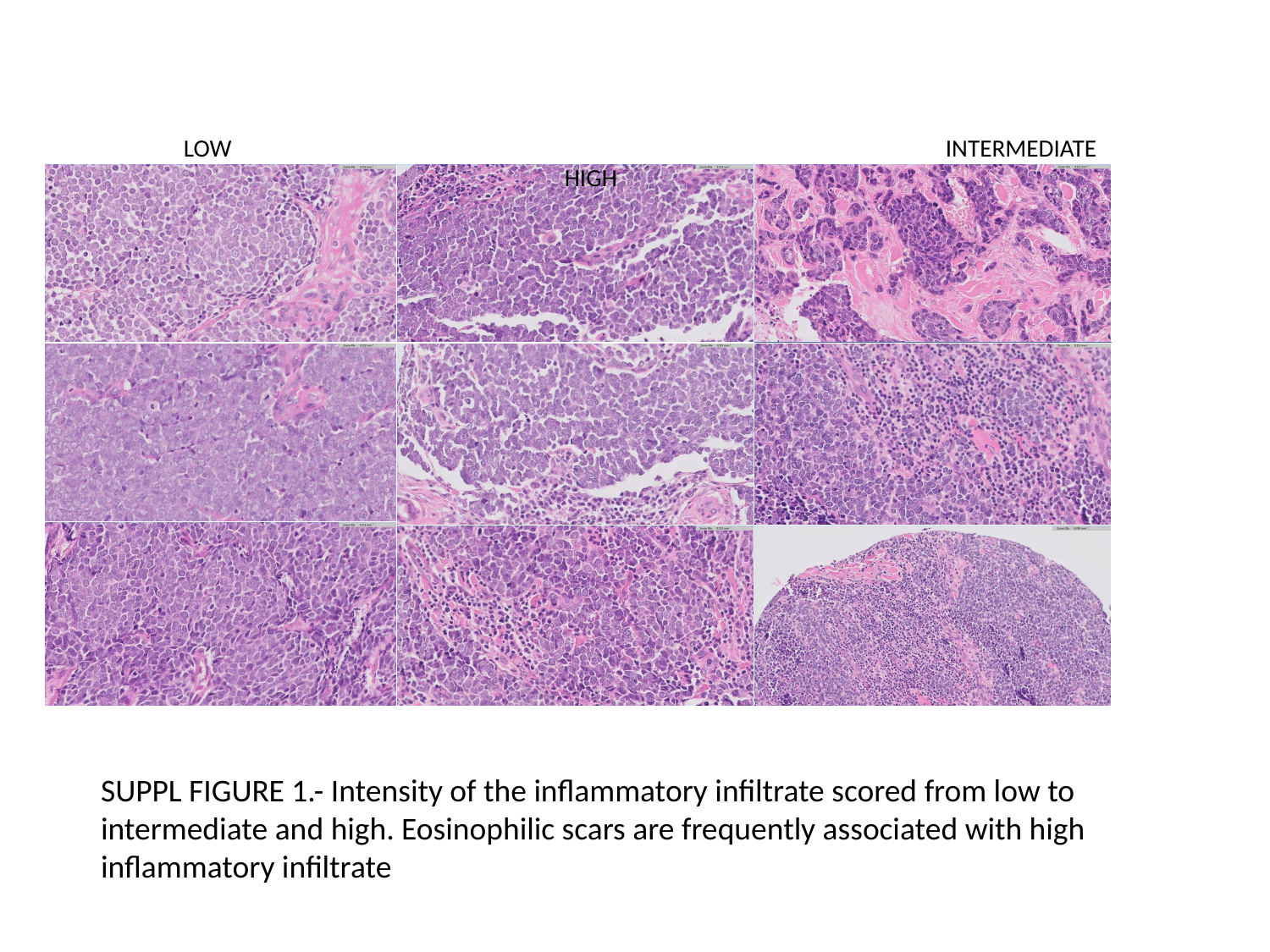

LOW						INTERMEDIATE				HIGH
SUPPL FIGURE 1.- Intensity of the inflammatory infiltrate scored from low to intermediate and high. Eosinophilic scars are frequently associated with high inflammatory infiltrate
